# Association of vision and hearing status with depressive symptoms among middle-aged and older Chinese adults

**DOI:** 10.3389/fpubh.2022.857307

**Published:** 2022-08-01

**Authors:** Yun-Guang Liu, Chao-Cai Wang, Qian Huang, Le Zhang, Yan Liu

**Affiliations:** ^1^Department of Public Health, Qinghai University Medical College, Xining, China; ^2^Department of Infection Disease, Qinghai Center for Disease Prevention and Control, Xining, China

**Keywords:** vision impairment, hearing impairment, depressive symptoms, older adults, China

## Abstract

**Objective:**

Long-term untreated vision and hearing impairments can negatively impact physical and mental wellbeing. We investigated the association of vision and hearing status with depressive symptoms among middle-aged and older Chinese adults.

**Methods:**

This was a prospective cohort study of 9,492 participants from the China Health and Retirement Longitudinal Study (CHARLS) carried out in 2011, 2013, 2015, and 2018. This study used self-reported vision and hearing status to determine the degree of impairment. Depressive symptoms were examined using the 10-item Center for Epidemiologic Studies Depression Scale (CESD-10), with a total score of ≥ 12 indicating depressive symptoms. A Cox proportional hazards model adjusted for age, sex, residence, marital status, educational level, smoking history, alcohol consumption, hypertension, diabetes, heart disease, digestive disease, arthritis, wearing glasses, and hearing aids was used to estimate the association of vision and hearing status with depressive symptoms among middle-aged and older Chinese adults.

**Results:**

Of the 9,492 participants [mean (SD) age at CHARLS baseline, 58.12 (9.00) years], 3,238 (34.11%) participants reported incident depressive symptoms during the 7-year follow-up period. Participants who self-reported only vision impairment [hazard ratios (HR): 1.14, 95% confidence intervals (CI): 1.05–1.24], only hearing impairment (HR: 1.24, 95% CI: 1.06–1.46), and both vision and hearing impairments (HR: 1.25, 95% CI: 1.08–1.45) were independently associated with a greater increase in the hazard risk of incident depressive symptoms compared to those without vision and hearing impairments. An increase in participants' vision and hearing scores was associated with a significant increase in the hazard risk of incident depressive symptoms (HR: 1.04, 95% CI: 1.03–1.06).

**Conclusion:**

Vision and hearing status was associated with increased depressive symptoms among middle-aged and older Chinese adults during the 7-year follow-up period. Participants' use of glasses and hearing aids did not improve their depressive symptoms. Our findings may facilitate the development of effective treatments to prevent and treat vision and hearing impairments, thereby enhancing the physical and mental wellbeing of middle-aged and older adults.

## Introduction

Vision and hearing impairments are common among middle-aged and older adults but are typically neglected or dismissed as a natural part of aging (1). The prevalence of vision and hearing impairments among middle-aged and older adults is high and is expected to further increase with the growth of the aging population (2, 3). The Global Burden of Disease (GBD) project results suggest that the prevalence of child and adult hearing impairment is substantially higher in middle- and low-income countries than in high-income countries, demonstrating the global need for attention to hearing impairment (4). According to a 2015 cross-sectional study, individuals' self-reported prevalence of vision and hearing impairments was higher in China than in European countries and the United States (5, 6). Despite the higher prevalence of vision and hearing impairments among middle-aged and older Chinese adults, most cases remain untreated. Accordingly, there is an urgent need to address the negative effects of vision and hearing impairments on physical and mental health.

Mental health among middle-aged and older adults often includes depressive symptoms, the risk of which increases with age (7). Indeed, previous studies estimated that ~20% of elderly individuals experience depressive symptoms (8). The prevalence of depressive symptoms among older adults with vision and/or hearing impairments is estimated to be 10–30%. Crucially, depressive symptoms may negatively impact the quality of life among elderly individuals (9). Most previous studies found that age, gender, location, chronic conditional diseases, physical activity, social isolation, IADL impairment, life satisfaction, and physical health are linked to depressive symptoms (10–12). Previous studies have indicated that vision and hearing impairments are of relevance in this regard, as vision and hearing-impaired older adults are more likely to develop depressive symptoms compared to those without vision and hearing impairments. Collectively, the literature suggests that vision and hearing impairments among older adults are associated with a higher risk of depressive symptoms compared to that among non-impaired older adults (5, 11, 12). Furthermore, the number of middle-aged and older adults with vision and/or hearing impairments in China is high. Notably, older adults with vision and/or hearing impairments receive insufficient medical care largely due to poor recognition, which highlights the need for adequate attention to be paid to this vulnerable population in conjunction with effective interventions.

Most previous studies that focused on the effects of vision and hearing impairments on depressive symptoms were conducted in developed countries and yielded mixed results. A systematic review reported the inability to perform a meta-analysis or establish a clear association between vision impairments and depressive symptoms in elderly individuals (13). Several cross-sectional studies have investigated the association of vision and hearing impairments with depressive symptoms among older Chinese adults (5). Nevertheless, no prospective cohort studies to date have examined the association of vision and hearing impairments with depressive symptoms in the elderly Chinese population. Accordingly, this study explored the hazard risk between vision and hearing status and depressive symptoms based on the China Health and Retirement Longitudinal Study (CHARLS) from 2011 (Wave 1) to 2018 (Wave 4). In this study, we hypothesized that: (1) compared to participants without vision and hearing impairment, participants with only vision impairment, only hearing impairment, and both vision and hearing impairment would have a significantly higher risk of depressive symptoms; (2) the hazard risk between vision and/or hearing impairments and depressive symptoms would persist even after adjusting for all risk factors (sociodemographic, lifestyle, and five types of chronic conditional diseases); (3) an increase in participants' vision and hearing scores would be associated with a greater increase in the risk of incident depressive symptoms among middle-aged and older adults; and (4) the effect size of wearing glasses and hearing aids would be implicated in the occurrence of depressive symptoms.

## Methods

### Data and participants

**Figure 1:**
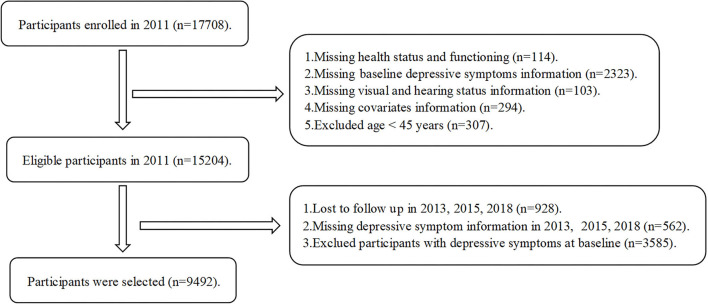
Flowchart of the selected participants from the CHARLS.

The CHARLS data utilized in this study were obtained in 2011 (Wave 1), 2013 (Wave 2), 2015 (Wave 3), and 2018 (Wave 4) (14). The CHARLS waves constituted a nationally representative longitudinal study that collected information regarding the demographic background, health status and functioning, and depressive symptoms of Chinese individuals aged 45 years and older. To ensure the representativeness of the participants, each wave employed a multistage probability sampling method to enroll individuals from 450 villages and 150 counties in 28 Chinese provinces. After the CHARLS 2011 baseline assessment, the CHARLS proceeded to follow-up with participants in three waves in 2013, 2015, and 2018. In response to deficiencies of incomplete baseline information in CHARLS, this study applied the following inclusion criteria: (1) participants aged 45 years or older and (2) participants not lost to follow-up. Furthermore, this study applied exclusion criteria as follows: (1) missing information on depressive symptoms at CHARLS 2011 baseline; (2) missing information on vision and hearing impairments at CHARLS 2011 baseline; (3) missing covariate information at CHARLS 2011 baseline; and (4) missing information on depressive symptoms during the 7-year follow-up. In this study, we used all data from 2011 (Wave 1), 2013 (Wave 2), 2015 (Wave 3), and 2018 (Wave 4). The final three waves were defined as three follow-up surveys during the 7-year follow-up period. This study was conducted in accordance with the Strengthening the Reporting of Observational Studies in Epidemiology (STROBE) reporting guidelines (15). This study included middle-aged and older adults aged 45 years and older. A list-wise deletion approach was used to treat for missing data, and this study excluded 8,416 participants. The final cohort included 9,492 participants at the CHARLS 2011 baseline. The detailed sampling selection process of the 9,492 participants with depressive symptoms included in the analysis in this prospective cohort study is presented in Figure 1. Furthermore, this study performed additional analyses to evaluate the robustness of our findings by adopting an inverse probability weighting (IPW) method to adjust for bias from non-random selection and missingness to obtain valid estimates in this study.

The CHARLS project was approved by the Peking University Biomedical Ethics Review Committee (IRB00001052-11015) (14). All participants provided written informed consent before participating in the study.

### Assessment of vision and hearing status

Assessment of vision and hearing impairments included three major questions based on participants' self-reported vision and hearing status from the CHARLS 2011 baseline. To evaluate participants' vision and hearing (6, 16), participants were asked the following questions: (1) “How good is your eyesight for seeing things at a distance, such as recognizing a friend from across the street?”; (2) “How good is your eyesight for seeing things up close, such as reading ordinary newspaper prints?”; and (3) “How good is your hearing?”. These three questions were evaluated on a five-point scale ranging from 1 to 5 (1 = excellent, 2 = very good, 3 = good, 4 = fair, and 5 = poor). Participants with vision impairments were defined as having poor eyesight for seeing things at a distance or up close, while participants with hearing impairments were defined as having poor hearing. Participants without vision and hearing impairments were defined as the absence of vision and hearing impairments. Conversely, participants with vision and hearing impairments were defined as having both vision and hearing impairments (5, 17, 18). To represent vision and hearing status, three types of independent variables were defined as follows: (1) the three questions included eyesight for seeing things at a distance (1 = excellent, 2 = very good, 3 = good, 4 = fair, and 5 = poor), eyesight for seeing things up close (1 = excellent, 2 = very good, 3 = good, 4 = fair, and 5 = poor), and hearing (1 = excellent, 2 = very good, 3 = good, 4 = fair, and 5 = poor); (2) vision and hearing impairments were divided into four groups (1 = no vision and hearing impairments, 2 = only vision impairments, 3 = only hearing impairments, and 4 = both vision and hearing impairments); and (3) three questions were combined to obtain vision and hearing scores (as a continuous variable) ranging from 3 to 15, with the highest score representing both vision and hearing impairments.

### Ascertainment of depressive symptoms

Depressive symptoms were treated as primary outcome events during follow-up. Participants were assessed using a 10-item short form of the Center for Epidemiologic Studies Depression Scale (CESD-10) in each CHARLS wave survey (19). Using the 10 items, participants were asked about their depressive feelings and behaviors in the previous week, such as whether they felt depressed, lonely, or fearful. Each question of the CESD-10 was evaluated on a four-point scale ranging from 0 to 3 [0 = <1 day (never or rarely), 1 = 1–2 days (some days), 2 = 3–4 days (occasionally), and 3 = 5–7 days (always)]. Before calculating the total 10-item scores, items 1, 2, 3, 4, 6, 7, 9, and 10 were scored, and reverse scoring was applied to items 5 and 8. Scale scores varied from 0 to 30, with a higher total score indicating a higher level of depressive symptoms. Depressive symptoms were defined as a binary dependent variable (1 = yes and 2 = no), with a total score of 12 indicating depressive symptoms (20, 21). The CESD-10 scale has been reported to have adequate reliability and validity among Chinese community-dwelling elderly individuals (17, 21).

### Covariate information assessment

Participants' covariate information at CHARLS 2011 baseline include (1) age (in years; 1 = 45–54, 2 = 55–64, 3 = 65–74, and 4 ≥ 75), sex (1 = male and 2 = female), residence (1 = rural and 0 = urban), education level (1 = illiterate, 2 = elementary school and below, 3 = middle school, and 4 = high school and above), marital status (1 = married/cohabiting and 2 = separated/divorced/widowed/never married), chronic diseases (1 = yes and 2 = no), smoking history (1 = yes and 2 = no), and alcohol consumption history (0 = none, 1 = drinks more than once a month, and 2 = drinks but less than once a month); (2) presence of chronic diseases, namely, hypertension (1 = yes and 0 = no), diabetes (1 = yes and 0 = no), heart disease (1 = yes and 0 = no), digestive disease (1 = yes and 0 = no), arthritis (1 = yes and 0 = no); and (3) self-assessed daily use of wearing glasses and/or a hearing aid (1 = yes and 0 = no). These risk factors were included as covariates in different models in this analysis.

### Statistical analyses

For dichotomous covariate information at the CHARLS 2011 baseline, we compared the differences among participants with and without depressive symptoms using a *t*-test or a chi-square test. A Cox proportional hazards model was used to calculate the hazard risk between vision and/or hearing status and depressive symptoms. Stratified analysis according to different characteristics was conducted to calculate the hazard risks between vision and hearing scores and depressive symptoms among participants, and the significance of all risk factor group interactions between vision and hearing scores and depressive symptoms was also calculated. The following four models were estimated in this study: Model 0 was unadjusted; Model 1 was adjusted for age, sex, residence, marital status, and educational level; Model 2 was adjusted for factors in Model 1 plus smoking history, alcohol consumption history, hypertension, diabetes, heart disease, digestive disease, and arthritis; and Model 3 was adjusted for factors in Model 2 plus wearing glasses and hearing aids. Covariate information was assumed to be missing at random in this study, and we performed a complete case analysis. The level of statistical significance was set at 95% (*P*-value < 0.05). Associations are presented as hazard ratios (HRs) with 95% confidence intervals (CIs). All data analyses were conducted using R statistical software version 4.1.2.

We performed two additional analyses to evaluate the robustness of our findings: (1) the Fine and Gray competing risk model to calculate the hazard risk of depressive symptoms and (2) the IPW method to adjust for bias from non-random selection and missingness to obtain valid estimates.

## Results

Of the 17,708 participants at CHARLS 2011 baseline, we excluded 114 participants with incomplete information on health status and functioning, 2,323 participants with incomplete information on depressive symptoms, 103 participants with missing vision and/or hearing status information, 294 participants with incomplete information on covariates, 307 participants younger than 45 years, 928 participants who were lost to follow-up, 562 participants without answers to the questions on depressive symptoms during the 7-year follow-up period, and 3,585 participants with depressive symptoms at CHARLS 2011 baseline. A final total of 9,492 participants were included in the analysis (Figure 1).

The average age of the 9,492 participants was 58.12 years (SD: 9.00 years). Of participants, 4,930 (51.94%) were men and 4,562 (48.06%) were women. The significance of the differences between participants with and without depressive symptoms stratified by different characteristics is presented in Table 1. Univariate analysis revealed that compared with those without incident depressive symptoms, participants with depressive symptoms were more likely to be older (57.98 ± 9.03 vs. 58.39 ± 8.93, *P-*value = 0.033), and significant differences were observed in gender (*P-*value < 0.001), residence (*P-*value < 0.001), education level (*P-*value < 0.001), marital status (*P-*value = 0.006), smoking history (*P-*value < 0.001), alcohol consumption (*P-*value < 0.001), heart disease (*P-*value = 0.046), digestive disease (*P-*value < 0.001), arthritis (*P-*value < 0.001), and wearing glasses (*P-*value = 0.045) among participants with incident depressive symptoms. Table 2 presents a comparison of the differences in CHARLS 2011 baseline characteristics between the included participants and those who were excluded from the analysis.

**Table 1:** The characteristics of the selected participants at CHARLS 2011 baseline.

**Characteristics**	**Participants, No. (%)**			***P*** **-value** ^a^
	**Total (*****N*** = **9,492)**	**Depressive symptoms** ^b^		
		**No (*****n*** = **6,254)**	**Yes (*****n*** = **3,238)**	
Age, years, mean (SD)	58.12 (9.00)	57.98 (9.03)	58.39 (8.94)	0.033
**Gender**				
Male	4,930 (51.94)	3,573 (57.13)	1,357 (41.91)	<0.001
Female	4,562 (48.06)	2,681 (42.87)	1,881 (58.09)	
**Residence**				
Urban	5,441 (57.32)	3,299 (52.75)	2,142 (66.15)	<0.001
Rural	4,051 (42.68)	2,955 (47.25)	1,096 (33.85)	
**Education level**				
Illiterate	2,107 (22.20)	1,142 (18.26)	965 (29.80)	<0.001
Elementary school and below	3,681 (38.78)	2,321 (37.11)	1,360 (42.00)	
Middle school	2,258 (23.79)	1,623 (25.95)	635 (19.61)	
High school and above	1,446 (15.23)	1,168 (18.68)	278 (8.59)	
**Marital status**				
Married/cohabiting	8,614 (90.75)	5,712 (91.33)	2,902 (89.62)	0.006
Separated/divorced /widowed/never married	878 (9.25)	542 (8.67)	336 (10.38)	
**Smoking history**				
Yes	3,915 (41.25)	2,766 (44.23)	1,149 (35.48)	<0.001
No	5,577 (58.75)	3,488 (55.77)	2,089 (64.52)	
**Alcohol consumption**				
Drink more than once a month	2,648 (27.48)	1,919 (30.68)	689 (21.18)	<0.001
Drink but less than once a month	787 (8.29)	546 (8.73)	241 (7.44)	
None of these	6,097 (64.23)	3,789 (60.59)	2,308 (71.28)	
**Hypertension**				
Yes	2,166 (22.82)	1,403 (22.43)	763 (23.56)	0.214
No	7,326 (77.18)	4,851 (77.57)	2,475 (76.44)	
**Diabetes**				
Yes	483 (5.09)	305 (4.88)	178 (5.50)	0.192
No	9,009 (94.91)	5,949 (95.12)	3,060 (94.50)	
**Heart disease**				
Yes	948 (9.99)	597 (9.55)	351 (10.84)	0.046
No	8,544 (90.01)	5,657 (90.45)	2,887 (89.16)	
**Digestive disease**				
Yes	1,779 (18.74)	1,059 (16.93)	720 (22.24)	<0.001
No	7,713 (81.26)	5,195 (83.07)	2,518 (77.76)	
**Arthritis**				
Yes	2,651 (27.93)	1,542 (24.66)	1,109 (34.25)	<0.001
No	6,841 (72.07)	4,712 (75.34)	2,129 (65.75)	
**Wearing glasses**				
Yes	1,098 (11.57)	753 (12.04)	345 (10.65)	0.045
No	8,394 (88.43)	5,501 (87.96)	2,893 (89.35)	
**Wearing hearing aids**				
Yes	46 (0.48)	30 (0.48)	16 (0.49)	0.924
No	9,446 (99.52)	6,224 (99.52)	3,222 (99.51)	

a *T-test or Pearson's χ^2^-test for the significance of the difference between participants with and without depressive symptoms*.

b *Defined as a score of 12 or greater on the 10-item Center for Epidemiologic Studies Depression scale*.

**Table 2:** The characteristics between participants included and not included at baseline.

**Characteristics**	**Not included**	**Included**	***P*** **-value**
Participants, No. (%)	8,216 (46.40)	9,492 (53.60)	
Age, years, mean (SD)	60.13 (11.25)	58.12 (9.00)	<0.001
**Gender, No (%)**			
Male	3,541 (43.80)	4,930 (58.20)	<0.001
Female	4,659 (50.53)	4,562 (49.47)	
**Residence, No (%)**			
Rural	3,117 (43.48)	4,051 (56.52)	<0.001
Urban	5,096 (48.36)	5,441 (51.64)	
**Education level, No (%)**			
Illiterate	2,696 (56.13)	2,107 (43.87)	<0.001
Elementary school and below	3,271 (47.05)	3,681 (52.95)	
Middle school	1,394 (38.17)	2,258 (61.83)	
High school and above	800 (35.62)	1,446 (64.38)	
**Marital status, No (%)**			
Married/cohabiting	6,803 (44.13)	8,614 (55.87)	<0.001
Separated/divorced /widowed/never married	1,380 (61.12)	878 (38.88)	
**Smoking history, No (%)**			
Yes	3,016 (43.51)	3,915 (56.49)	<0.001
No	5,051 (47.53)	5,577 (52.47)	
**Alcohol consumption, No (%)**			
Drink more than once a month	1,775 (40.50)	2,608 (59.50)	<0.001
Drink but less than once a month	597 (43.14)	787 (56.86)	
None of these	5,688 (58.26)	6,097 (51.74)	
**Hypertension, No (%)**			
Yes	2,118 (49.44)	2,166 (50.56)	<0.001
No	5,860 (44.44)	7,326 (55.56)	
**Diabetes, No (%)**			
Yes	510 (51.36)	483 (48.64)	<0.001
No	7406 (45.12)	9009 (54.88)	
**Heart disease, No (%)**			
Yes	1,145 (54.71)	948 (45.29)	<0.001
No	6,830 (44.43)	8,544 (55.67)	
**Digestive disease, No (%)**			
Yes	2,123 (54.41)	1,779 (45.59)	<0.001
No	5,902 (43.35)	7,713 (56.65)	
**Arthritis, No (%)**			
Yes	3,122 (54.08)	2,651 (45.92)	<0.001
No	4,910 (41.78)	6,841 (58.22)	
**Wearing glasses, No (%)**			
Yes	870 (44.21)	1,098 (55.79)	0.184
No	7,091 (45.79)	8,394 (54.21)	
**Wearing hearing aid, No (%)**			
Yes	52 (53.06)	46 (46.94)	0.158
No	8,024 (45.93)	9,446 (54.07)	

At CHARLS 2011 baseline, 6,463 (68.09%) participants had no vision and hearing impairments, 2,154 (22.69%) had only vision impairments, 407 (4.29%) had only hearing impairments, and 468 (4.93%) had both vision and hearing impairments. After a 7-year follow-up period, 3,238 (34.11%) participants reported incident depressive symptoms. Table 3 presents the association of vision and hearing status with depressive symptoms and the effect size of wearing glasses and hearing aids on depressive symptoms. After adjusting for potential covariates (in Model 3), a comparison of poor with excellent revealed adjusted HRs of 1.59 (95% CI: 1.16–2.17) for eyesight for seeing things at a distance, 1.28 (95% CI: 0.91–1.83) for eyesight for seeing things up close, and 1.42 (95% CI: 1.02–1.98) for hearing. Compared with no vision and hearing impairments, only vision impairments (HR: 1.13, 95% CI: 1.04–1.22), only hearing impairments (HR: 1.20, 95% CI: 1.02–1.41), and both vision and hearing impairments (HR: 1.21, 95% CI: 1.05–1.41) were significantly associated with depressive symptoms. The hazard risk of depressive symptoms increased with an improvement in vision and hearing scores (HR: 1.04, 95% CI: 1.02–1.06). Furthermore, compared with participants that did not wear glasses and hearing aids, participants that wore glasses (HR: 1.02, 95% CI: 0.91–1.15) and hearing aids (HR: 1.25, 95% CI: 0.76–2.05) did not dismiss the association.

**Table 3:** Association of vision and hearing status with depressive symptoms among middle-aged and older Chinese adults.

**Vision and hearing status (No.)**	**Outcome case, No (%)**	**Depressive symptom**^e^ **HR (95% CI)**			
		**Model 0** ^a^	**Model 1** ^b^	**Model 2** ^c^	**Model 3** ^d^
**Eyesight for seeing things at a distance**					
Excellent (189)	43 (22.75)	1.00 (Reference)	1.00 (Reference)	1.00 (Reference)	1.00 (Reference)
Very good (1,413)	376 (26.61)	1.12 (0.87–1.64)	1.15 (0.84–1.57)	1.15 (0.84–1.58)	1.15 (0.84–1.58)
Good (2,693)	894 (33.20)	1.56 (1.14–2.11)^**^	1.39 (1.02–1.89)^*^	1.36 (1.01–1.86)^*^	1.37 (1.01–1.86)^*^
Fair (3,732)	1,303 (34.91)	1.66 (1.22–2.24)^**^	1.43 (1.05–1.94)^*^	1.37 (1.02–1.87)^*^	1.38 (1.02–1.87)^*^
Poor (1,465)	622 (42.46)	2.15 (1.57–2.93)^***^	1.71 (1.25–2.33)^***^	1.59 (1.17–2.17)^**^	1.59 (1.16–2.17)^**^
**Eyesight for seeing things up close**					
Excellent (132)	33 (25.00)	1.00 (Reference)	1.00 (Reference)	1.00 (Reference)	1.00 (Reference)
Very good (978)	293 (29.96)	1.24 (0.87–1.78)	1.19 (0.83–1.71)	1.20 (0.84–1.73)	1.20 (0.84–1.72)
Good (2,470)	827 (33.48)	1.42 (1.00–2.01)	1.30 (0.91–1.84)	1.27 (0.89–1.80)	1.27 (0.89–1.79)
Fair (4,085)	1,371 (33.56)	1.43 (1.01–2.02)^*^	1.25 (0.89–1.77)	1.21 (0.86–1.71)	1.21 (0.86–1.71)
Poor (1,827)	714 (39.08)	1.59 (1.73–2.46)^**^	1.47 (1.04–2.09)^*^	1.39 (0.98–1.98)	1.39 (0.91–1.97)
**Hearing**					
Excellent (158)	40 (25.31)	1.00 (Reference)	1.00 (Reference)	1.00 (Reference)	1.00 (Reference)
Very good (1,534)	438 (28.55)	1.15 (0.83–1.59)	1.10 (0.80–1.53)	1.10 (0.80–1.53)	1.10 (0.80–1.52)
Good (3,221)	1,042 (32.35)	1.33 (0.97–1.82)	1.22 (0.89–1.67)	1.20 (0.88–1.65)	1.20 (0.88–1.65)
Fair (3,704)	1,355 (36.58)	1.56 (1.14–2.13)^**^	1.40 (1.02–1.91)^*^	1.33 (0.97–1.83)	1.33 (0.97–1.83)
Poor (875)	363 (41.49)	1.82 (1.31–2.53)^***^	1.58 (1.14–2.20)^**^	1.50 (1.08–2.08)^*^	1.49 (1.07–2.07)^*^
**Vision and hearing impairment group**					
No vision and hearing impairment (6,463)	2,043 (31.61)	1.00 (Reference)	1.00 (Reference)	1.00 (Reference)	1.00 (Reference)
Only vision impairment (2,154)	832 (38.62)	1.30 (1.19–1.40)^***^	1.18 (1.09–1.28)^***^	1.14 (1.05–1.24)^**^	1.14 (1.05–1.24)^**^
Only hearing impairment (407)	161 (40.15)	1.31 (1.12–1.54)^***^	1.27 (1.08–1.49)^**^	1.25 (1.06–1.47)^**^	1.24 (1.06–1.46)^**^
Both vision and hearing impairment (468)	202 (43.16)	1.49 (1.29–1.73)^***^	1.33 (1.15–1.54)^***^	1.25 (1.08–1.45)^**^	1.25 (1.08–1.45)^**^
**Risk factors** ^f^					
Age			1.00 (0.99–1.00)	1.00 (0.99–1.00)	1.00 (0.99–1.00)
**Gender**					
Male			1.00 (Reference)	1.00 (Reference)	1.00 (Reference)
Female			1.52 (1.41–1.64)^***^	1.42 (1.28–1.58)^***^	1.42 (1.28–1.58)^***^
**Residence**					
Urban			1.00 (Reference)	1.00 (Reference)	1.00 (Reference)
Rural			0.71 (0.66–0.76)^***^	0.71 (0.66–0.76)^***^	0.71 (0.65–0.76)^***^
**Education level**					
Illiterate			1.00 (Reference)	1.00 (Reference)	1.00 (Reference)
Elementary school and below			0.92 (0.84–1.00)	0.91 (0.83–0.99)^*^	0.90 (0.83–0.99)^*^
Middle school			0.71 (0.63–0.79)^***^	0.71 (0.64–0.80)^***^	0.71 (0.63–0.79)^***^
High school and above			0.50 (0.43–0.57)^***^	0.50 (0.43–0.57)^***^	0.49 (0.43–0.57)^***^
**Marital status**					
Married/cohabiting			1.00 (Reference)	1.00 (Reference)	1.00 (Reference)
Separated/divorced/widowed/never married			1.04 (0.92–1.17)	1.04 (0.93–1.18)	1.04 (0.93–1.18)
**Smoking history**					
Yes				1.05 (0.95–1.16)	1.05 (0.95–1.16)
No				1.00 (Reference)	1.00 (Reference)
**Alcohol consumption**					
Drink more than once a month				1.15 (1.00–1.34)	1.15 (0.99–1.33)
Drink but less than once a month				1.22 (1.10–1.34)^***^	1.22 (1.10–1.34)^***^
None of these				1.00 (Reference)	1.00 (Reference)
**Hypertension**					
Yes				1.03 (0.95–1.13)	1.03 (0.95–1.13)
No				1.00 (Reference)	1.00 (Reference)
**Diabetes**					
Yes				1.21 (1.03–1.41)^*^	1.21 (1.03–1.41)^*^
No				1.00 (Reference)	1.00 (Reference)
**Heart disease**					
Yes				1.07 (0.96–1.21)	1.07 (0.96–1.21)
No				1.00 (Reference)	1.00 (Reference)
**Digestive disease**					
Yes				1.21 (1.11–1.32)^***^	1.21 (1.11–1.31)^***^
No				1.00 (Reference)	1.00 (Reference)
**Arthritis**					
Yes				1.29 (1.20–1.39)^***^	1.29 (1.20–1.39)^***^
No				1.00 (Reference)	1.00 (Reference)
**Wearing glasses**					
Yes					1.02 (0.91–1.15)
No					1.00 (Reference)
**Wearing hearing aids**					
Yes					1.25 (0.76–2.05)
No					1.00 (Reference)

*HR, hazard ratio; CI, confidence interval*.

* *0.01 < P-value < 0.05,*

** *0.001 < P-value < 0.01,*

*** *P-value < 0.001*.

a *Model 0 was unadjusted*.

b *Model 1 was adjusted for age, sex, residence, marital status, and educational level*.

c *Model 2 was adjusted for age, sex, residence, marital status, educational level, smoking history, alcohol consumption, hypertension, diabetes, heart disease, digestive disease, and arthritis*.

d *Model 3 was adjusted for age, sex, residence, marital status, educational level, smoking history, alcohol consumption, hypertension, diabetes, heart disease, digestive disease, arthritis, wearing glasses, and wearing hearing aids*.

e *Defined as a score of 12 or greater on the 10-item Center for Epidemiologic Studies Depression scale*.

f *Hazard ratio on the risk factors of vision and hearing impairment group*.

**Figure 2:**
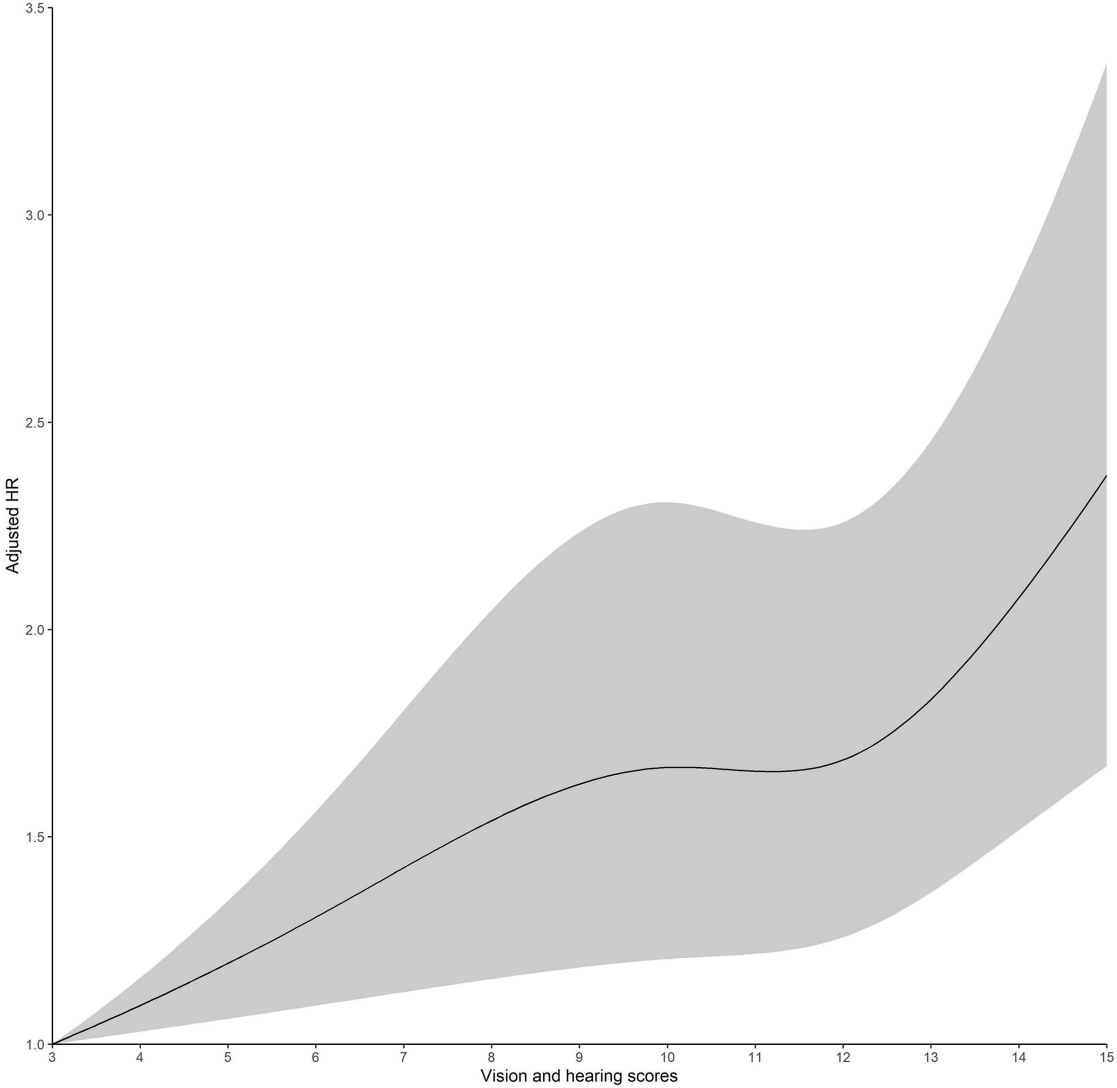
Adjusted hazard ratios (HRs) of depressive symptoms, according to vision and hearing scores. Graphs show HRs for depressive symptoms adjusted for age, sex, residence, marital status, educational level, smoking history, alcohol consumption, hypertension, diabetes, heart disease, digestive disease, arthritis, wearing glasses, and wearing a hearing aid. Data were fitted by a restricted cubic spline Cox proportional hazards regression model. The vision and hearing scores range from 3 to 15, with the highest score representing impairment of vision and hearing. Solid lines indicate HRs, and dashed lines indicate 95% CIs.

**Figure 3:**
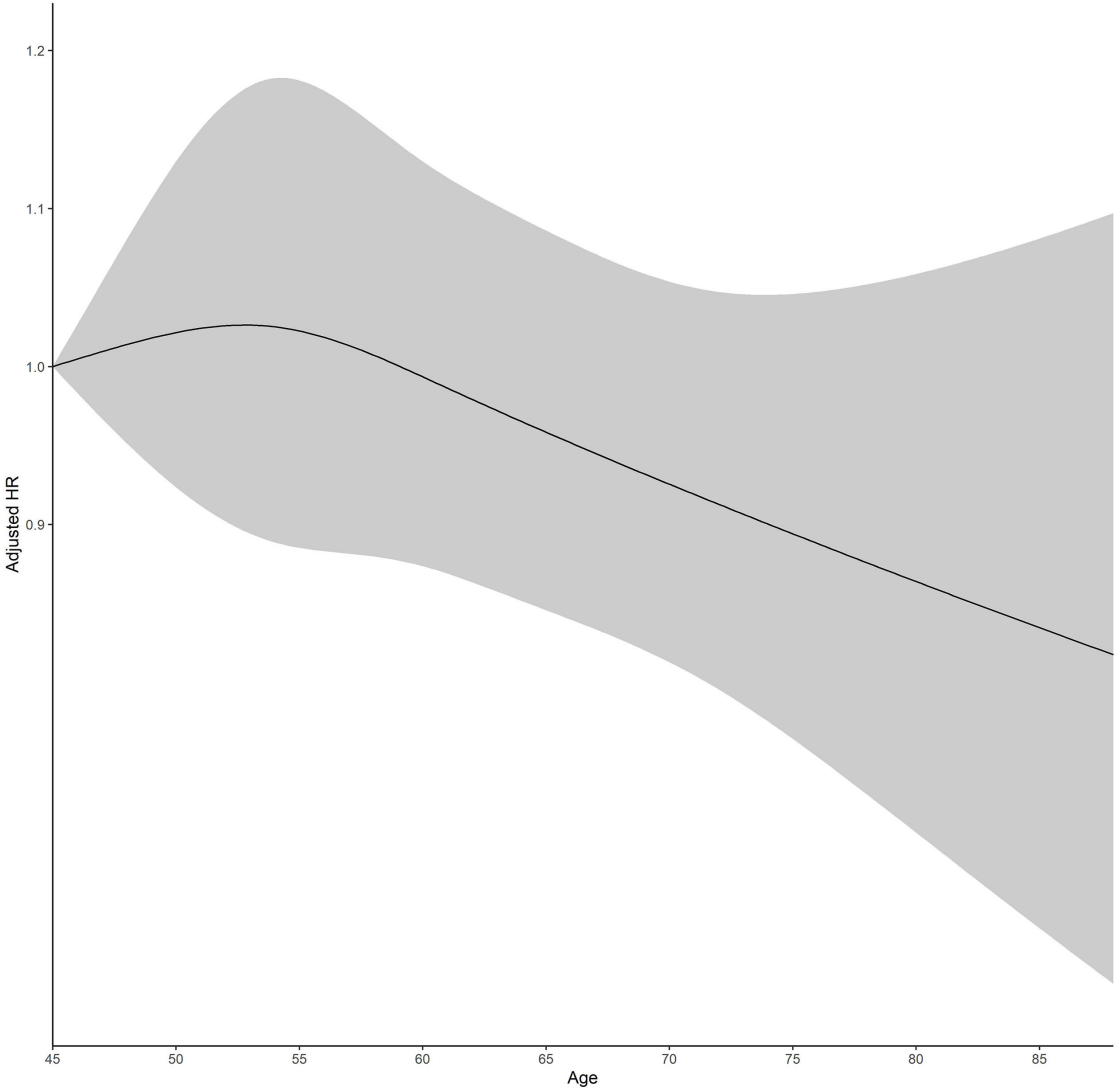
Adjusted hazard ratios (HRs) of depressive symptoms, according to age. Graphs show HRs for depressive symptoms adjusted for vision and hearing scores, sex, residence, marital status, educational level, smoking history, alcohol consumption, hypertension, diabetes, heart disease, digestive disease, arthritis, wearing glasses, and wearing a hearing aid. Data were fitted by a restricted cubic spline Cox proportional hazards regression model. The vision and hearing scores range from 45 to 85. Solid lines indicate HRs, and dashed lines indicate 95% CIs.

Restricted cubic spline regression revealed a positive linear association between vision and hearing scores and the hazard risk of depressive symptoms (for non-linearity: χ^2^*-*value = 7.30, *P-*value = 0.026) (Figure 2) and a positive linear association between age and the hazard risk of depressive symptoms (for non-linearity: χ^2^*-*value = 1.22, *P-*value = 0.545) (Figure 3).

There was no evidence that our results lacked robustness in the two additional analyses. Table 4 presents similar findings between the Fine and Gray competing risk model and Cox proportional hazards model in Table 3. Table 5 presents the association of vision and hearing scores with depressive symptoms before using the Cox proportional hazards model (HR: 1.05, 95% CI: 1.03–1.06) and after using the IPW method (HR: 1.93, 95% CI: 1.75–2.12).

**Table 4:** Association of vision and hearing status with depressive symptoms by the Fine and Gray competing risk analysis.

**Vision and hearing status**	**Depressive symptom status**^b^ **HR (95% CI)**
	**Model** ^a^
**Eyesight for seeing things at a distance**	
Excellent	1.00 (Reference)
Very good	1.14 (0.84–1.54)
Good	1.34 (1.00–1.18)
Fair	1.35 (1.01–1.80)^*^
Poor	1.54 (1.15–2.07)^**^
**Eyesight for seeing things up close**	
Excellent	1.00 (Reference)
Very good	1.19 (0.85–1.67)
Good	1.25 (0.91–1.73)
Fair	1.20 (0.87–1.66)
Poor	1.36 (0.99–1.89)
**Hearing**	
Excellent	1.00 (Reference)
Very good	1.09 (0.81–1.49)
Good	1.19 (0.88–1.60)
Fair	1.31 (0.97–1.76)
Poor	1.44 (1.06–1.97)^*^
**Vision and hearing impairment group**	
No vision and hearing impairment	1.00 (Reference)
Only vision impairment	1.13 (1.05–1.22)^***^
Only hearing impairment	1.22 (1.05–1.41)^***^
Both vision and hearing impairment	1.23 (1.07–1.40)^***^

*HR, hazard ratio; CI, confidence interval*.

* *0.01 < P-value < 0.05,*

** *0.001 < P-value < 0.01,*

*** *P-value < 0.001*.

a *Model was adjusted for age, sex, residence, marital status, educational level, smoking history, alcohol consumption, hypertension, diabetes, heart disease, digestive disease, arthritis, wearing glasses, and wearing hearing aids*.

b *Defined as a score of 12 or greater on the 10-item Center for Epidemiologic Studies Depression scale*.

**Table 5:** Associations between vision and hearing scores and depressive symptoms by Cox and IPW methods.

**Methods**	**Depressive symptom status**^b^ **HR (95% CI)**
	**Model** ^a^
Vision and/or hearing score (Cox)	1.05 (1.03–1.06)^***^
Vision and/or hearing score (IPW)	1.93 (1.75–2.12)^***^

*HR, Hazard Ratio; CI, Confidence Interval; Cox, Cox proportional hazards model; IPW, Inverse probability weighting*.

*** *P-value < 0.001*.

a *Model was adjusted for age, sex, residence, marital status, educational level, smoking history, alcohol consumption, hypertension, diabetes, heart disease, digestive disease, arthritis, wearing glasses, and wearing hearing aids*.

b *Defined as a score of 12 or greater on the 10-item Center for Epidemiologic Studies Depression scale*.

Table 6 presents the association of vision and hearing impairments with depressive symptoms, stratified by different risk factors. No significant interactions were noted among risk factors (all *P-*values for interaction > 0.05). The association of vision and hearing scores with depressive symptoms differed among participants stratified according to different risk factors at baseline. The analysis stratified according to different risk factors revealed that participants with depressive symptoms were more likely to be younger, had a higher level of education, did not consume alcohol, and did not wear glasses or hearing aids.

**Table 6:** Association of vision and hearing scores with depressive symptoms stratified by different risk factors using Cox.

**Characteristics**	**Depressive symptom**^b^ **HR (95% CI)**	***P*** **-value**	***P*** **-value for interaction**
**Overall**	1.05 (1.03–1.06)	<0.001	
**Age (years)**			0.735
45–54	1.06 (1.03–1.08)	0.001	
55–64	1.03 (1.00–1.06)	0.037	
65–74	1.07 (1.03–1.11)	<0.001	
≥75	1.06 (0.98–1.14)	0.128	
**Gender**			0.484
Male	1.05 (1.02–1.08)	<0.001	
Female	1.04 (1.02–1.07)	<0.001	
**Residence**			0.853
Urban	1.04 (1.02–1.06)	<0.001	
Rural	1.05 (1.02–1.08)	<0.001	
**Education level**			0.272
Illiterate	1.03 (1.00–1.06)	0.082	
Elementary school and below	1.05 (1.02–1.07)	0.001	
Middle school	1.06 (1.02–1.10)	0.004	
High school and above	1.09 (1.03–1.16)	0.002	
**Marital status**			0.751
Married/cohabiting	1.04 (1.03–1.06)	<0.001	
Separated/divorced/ widowed/ never married	1.06 (1.01–1.11)	0.026	
**Smoking history**			0.825
Yes	1.05 (1.02–1.08)	<0.001	
No	1.05 (1.02–1.07)	<0.001	
**Alcohol consumption**			0.294
Drink more than once a month	1.03 (0.99–1.06)	0.137	
Drink but less than once a month	1.09 (1.03–1.16)	0.005	
None of these	1.05 (1.03–1.07)	<0.001	
**Hypertension**			0.866
Yes	1.06 (1.02–1.09)	0.003	
No	1.04 (1.02–1.06)	<0.001	
**Diabetes**			0.107
Yes	1.10 (1.01–1.19)	0.022	
No	1.04 (1.03–1.06)	<0.001	
**Heart disease**			0.111
Yes	1.02 (0.96–1.07)	0.540	
No	1.05 (1.03–1.07)	<0.001	
**Digestive disease**			0.892
Yes	1.04 (1.01–1.08)	0.020	
No	1.05 (1.01–1.07)	<0.001	
Arthritis			0.134
Yes	1.03 (1.00–1.07)	0.029	
No	1.05 (1.03–1.07)	<0.001	
Wearing glasses			0.909
Yes	1.05 (0.99–1.10)	0.101	
No	1.05 (1.03–1.06)	<0.001	
Wearing hearing aids			0.284
Yes	0.79 (0.52–1.19)	0.253	
No	1.05 (1.03–1.07)	<0.001	

*HR, hazard ratio; CI, confidence interval*.

^a^Model was adjusted for age, sex, residence, marital status, educational level, smoking history, alcohol consumption, hypertension, diabetes, heart disease, digestive disease, arthritis, wearing glasses, and wearing hearing aids.

b *Defined as a score of 12 or greater on the 10-item Center for Epidemiologic Studies Depression scale*.

## Discussion

This study established the association between vision and hearing status and depressive symptoms in middle-aged and older Chinese adults. Our findings provide evidence based on a nationally representative cohort study sample of 9,492 Chinese community-dwelling elderly individuals and longitudinal follow-up to support our hypotheses. This study had four major findings based on the cohort study data. First, participants with only vision impairments, only hearing impairments, and both vision and hearing impairments exhibited incident depressive symptoms during the 7-year follow-up period. Second, the fact that all risk factors (e.g., age, sex, chronic diseases, and lifestyle) were obtained using CHARLS self-reported questionnaires does not diminish the interpretation of the association between vision and/or hearing status and depressive symptoms. Third, participants with higher vision and hearing scores had a greater risk of depressive symptoms. Finally, the use of glasses and hearing aids did not improve depressive symptoms. In addition, this study excluded 8,416 participants from the CHARLS baseline, which may have contributed to the non-representativeness of the 9,492 participants included in this study and the acquisition of erroneous estimates. Therefore, this study used the IPW method to adjust for bias from non-random selection and missingness to obtain valid estimates of the association.

Our findings identified a total of 2,154 (22.69%) participants with only vision impairments, 407 (4.29%) with only hearing impairments, and 468 (4.93%) with both vision and hearing impairments at the CHARLS 2011 baseline. After the 7-year follow-up period, 3,238 (34.11%) participants reported incident depressive symptoms. Our findings revealed that higher vision and hearing scores were indicative of more depressive symptoms. As reported by Armenia (11), the prevalence of vision impairments (moderate and severe poor vision) was 13.3%, and almost 3.8% of patients were blind. A study on Italian elderly individuals revealed that the incidence of vision impairments was 1.4% and the prevalence of hearing impairments was as low as 0.2% but increased with age (22). A survey conducted in four typical Chinese provinces reported that over two-thirds of Chinese adults aged 60 years and older had hearing impairments (23). Moreover, a study in Singapore reported that patients with glaucoma often presented with coexisting psychiatric disorders such as anxiety and depression. And there was a relatively high prevalence of depression (30%) and anxiety disorder (64%) among glaucoma patients in Singapore; notably, female patients with glaucoma were more likely to present with depressive symptoms (24). Our findings revealed that the prevalence of vision and hearing impairments in China was distinct from that reported in other studies, which could be due to the different assessment methods and characteristics of the populations involved in the studies performed in different countries.

Our results revealed that participants with only vision impairments, only hearing impairments, and both vision and hearing impairments presented with depressive symptoms but with a different degree of risk. Several population-based longitudinal studies in Canada (25), the Salisbury Eye Evaluation Study (26), and the National Health and Aging Trends Study (27) confirmed a link between depressive symptoms and vision impairments. A prevailing concern among hearing-impaired individuals in China is the impact of hearing impairments on physical and mental health (23, 28). Indeed, the Korean health insurance cohort study (29) reported that severe hearing impairments significantly increased the risk of depressive symptoms in all age groups of the study population after matching for age, sex, income, and region. A population-based longitudinal study reported that vision and hearing impairments were significantly associated with depressive symptoms and suggested that treating or rehabilitating either vision or hearing impairments may incident depressive symptoms (25, 30, 31). According to several prospective studies, the presence of depressive symptoms is associated with reduced sensory function, and the risk of depressive symptoms increases over subsequent years (28, 32). A multi-site cohort study of community-dwelling persons aged 65 years and over from the electoral rolls of three French cities suggested that this relationship is complex depending on whether the loss is related to near visual impairment or visual function loss and visual decline across time (33). Consistent with previous studies, our findings also demonstrated that an increase in vision and hearing scores was associated with a higher risk of depressive symptoms after adjusting for potential confounders, and further studies are warranted to clarify this dose–effect relationship. However, it should be noted that CESD-10 is different from clinical measures of depressive symptoms, and these results should be interpreted with caution among middle-aged and older adults.

Consistent with previous studies, our findings indicated that participants with depressive symptoms stratified by potential risk factors were more likely to be younger, have a high level of education, not consume alcohol, and not wear glasses and hearing aids. Our data revealed that age was not a significant risk factor for this association. Depression is a serious public health concern among elderly individuals; it causes disability, medical illnesses, mental health problems, and increased mortality (11). Previous research has reported that individuals with vision and/or hearing impairments have a higher risk of depressive symptoms (2, 34). In addition to vision and hearing impairments, previous studies found that older age, female sex, lower education, illness, mobility, IADL, sleep time, smoking, residential setting, emotional support, negative interactions with family members, social participation, and physical activities are more likely to be associated with depressive symptoms (6, 30, 35). As participants' chronic diseases were self-reported, the results obtained in this stratified analysis should not be used to infer causal associations between chronic diseases and depressive symptoms. Nevertheless, we observed an increased likelihood of depression with age. Of note, a higher education level did not reduce the occurrence of depressive symptoms, which may be because individuals with higher levels of education have more risk factors influencing their mental health.

In this study, vision and hearing status was self-reported in CHARLS. In this regard, objective clinical vision and hearing screening are more appropriate than subjective self-reported vision and hearing status, although a previous study reported the opposite result (36). Consistent with existing literature, our findings suggest longitudinal trends in self-reported vision impairments and depressive symptoms. Clinical vision and hearing screening may be more appropriate than self-reported vision and hearing impairment status to more accurately assess vision and hearing status in older adults. A prospective study employed objective hearing tests to assess the relationship between hearing impairments and depressive symptoms (29). Results of the Georgia Centenarian Study suggest that objective vision status does not always reflect subjective status, and various factors affect the relationship between objective vision function and self-reported vision problems (37). Previous studies have predominantly focused on self-reported vision and hearing status as a key indicator of actual vision and hearing impairments in elderly individuals. Some studies have compared the two vision measures regarding mental health and reported that self-reported vision function loss was a better predictor of depression compared to the indicators of objective vision acuity (38, 39). Previous research comparing vision measurements in relation to mental health has indicated that self-reported vision functional impairments were a stronger predictor of depressive symptoms compared to objective measures of vision acuity (38). In addition, a systematic review suggested the inability to conduct a meta-analysis or establish an association between vision impairments and depressive symptoms in elderly individuals for various reasons, namely, a lack of standardized vision impairment measures, different depressive symptom scales, comparability of the studies, and the potential bias caused by different variables (13). Consistent with previous studies, our findings demonstrated that subjective self-reported vision and hearing impairments were associated with depressive symptoms among middle-aged and older adults. Future research should examine objective measures and subjective self-reported disparities in vision and hearing impairments in China.

In this study, only a small number of participants wore glasses and hearing aids. Moreover, the use of glasses and hearing aids did not attenuate the association of vision and hearing impairments with depressive symptoms. The use of glasses and hearing aids is lower in older Chinese adults, which may be modulated by outdated beliefs (e.g., wearing glasses or hearing aids can lead to further impairments in vision or hearing) (6). Furthermore, older adults with vision and hearing impairments who are treated with glasses, hearing aids, and other therapeutic approaches exhibit improvements in depressive symptoms, wellbeing, and quality of life (6, 22, 40). A population-based longitudinal study reported that vision impairments were significantly associated with depressive symptoms and suggested that the treatment or rehabilitation of vision impairments could help to prevent depressive symptoms (30). Similarly, other studies have suggested that vision impairments in elderly individuals can increase their risk of developing depressive symptoms. Accordingly, early interventions to prevent the occurrence and onset of vision impairments are warranted to improve the quality of life of these individuals (41, 42). The severity of hearing impairments is closely related to multiple physical and mental health outcomes among middle-aged and older Chinese adults, and actions should be taken to prevent and treat hearing impairments to improve health and wellbeing (28, 32). In this regard, the use of glasses and hearing aids to improve depressive symptoms is noteworthy, and medical and health education initiatives for older adults with vision and hearing impairments are warranted.

The strengths of our research design based on a large population in China and distinct vision and hearing analyses revealed a significant connection between vision and hearing status and depressive symptoms, which was based on the CHARLS design of a prospective cohort study with a 7-year follow-up period. However, this study has several limitations. First, additional confounding factors linked with depressive symptoms, such as income, life satisfaction, and social support, were not included as covariates for model adjustment. Second, vision and hearing status was self-reported and was not based on medical records in CHARLS. Third, only a small number of participants wore glasses and hearing aids; hence, any improvements could not be conclusively determined. Participants were only asked about their vision and hearing impairments and use of glasses and hearing aids at baseline but not during the 7-year follow-up period, during which we focused only on depressive symptoms. Finally, the self-reported variables may have introduced recall bias when the study included older adults in CHARLS.

In conclusion, this study demonstrated that vision and hearing impairments were associated with depressive symptoms among middle-aged and older Chinese adults. Furthermore, our findings revealed that participants with only vision impairments, only hearing impairments, and both vision and hearing impairments were at a higher risk of incident depressive symptoms. Based on our findings, individuals with both vision and hearing impairments had the highest risk of developing depressive symptoms, followed by those with only hearing or vision impairments. The presence of other risk factors did not affect the interpretation of the association of vision and hearing impairments with depressive symptoms. Moreover, an increase in participants' vision and hearing scores was associated with an increase in the hazard risk of incident depressive symptoms among middle-aged and older Chinese adults. In addition, participants' use of glasses and hearing aids did not improve depressive symptoms. Despite the growing burden of vision and hearing impairments in China's aging society, these conditions have been overlooked. Accordingly, effective treatments to prevent and treat vision and hearing impairments are warranted to improve the mental health and quality of life of middle-aged and older individuals with vision and hearing impairments.

## Data availability statement

Publicly available datasets were analyzed in this study. This data can be found here: http://charls.pku.edu.cn/.

## Ethics statement

The studies involving human participants were reviewed and approved by Peking University Biomedical Ethics Review Committee (IRB00001052-11015). The patients/participants provided their written informed consent to participate in this study.

## Author contributions

Y-GL performed data analysis, interpreted the data, and drafted the manuscript. C-CW interpreted the data and reviewed the manuscript. QH and LZ performed data collection and data cleaning. YL designed the study, reviewed, and critically revised the manuscript. All authors contributed to the article and approved the submitted version.

## Funding

This study was supported by the Qinghai province high-end innovative talents thousand talents program (No. 2020-12) and Graduate Program Construction Project of Qinghai University (Project Number: qdyk-200301). The funders played no role in designing or conducting the study or in the collection, management, analysis, or interpretation of the data, nor did they have any input into preparation, review, or approval of this manuscript.

## Conflict of interest

The authors declare that the research was conducted in the absence of any commercial or financial relationships that could be construed as a potential conflict of interest.

## Publisher's note

All claims expressed in this article are solely those of the authors and do not necessarily represent those of their affiliated organizations, or those of the publisher, the editors and the reviewers. Any product that may be evaluated in this article, or claim that may be made by its manufacturer, is not guaranteed or endorsed by the publisher.

## References

[1] Gopinath B Liew G Burlutsky G McMahon CM Mitchell P. Association between vision and hearing impairment and successful aging over five years. Maturitas. (2021) 143:203–8. 10.1016/j.maturitas.2020.10.015 33308630

[2] Brunes A Heir T. Visual impairment and depression: age-specific prevalence, associations with vision loss, and relation to life satisfaction. World J Psychiatry. (2020) 10:139–49. 10.5498/wjp.v10.i6.139 32742947 PMC7360524

[3] Bourne RRA Flaxman SR Braithwaite T Cicinelli MV Das A Jonas JB. Magnitude, temporal trends, and projections of the global prevalence of blindness and distance and near vision impairment: a systematic review and meta-analysis. Lancet Glob Health. (2017) 5:e888–97. 10.1016/S2214-109X(17)30293-0 28779882

[4] Stevens G Flaxman S Brunskill E Mascarenhas M Mathers CD Finucane M. Global and regional hearing impairment prevalence: an analysis of 42 studies in 29 countries. Eur J Public Health. (2013) 23:146–52. 10.1093/eurpub/ckr176 22197756

[5] Rong H Lai X Jing R Wang X Fang H Mahmoudi E. Association of sensory impairments with cognitive decline and depression among older adults in China. JAMA Netw Open. (2020) 3:e2014186. 10.1001/jamanetworkopen.2020.14186 32990739 PMC7525357

[6] Heine C Browning CJ Gong CH. Sensory loss in China: prevalence, use of aids, and impacts on social participation. Front Public Health. (2019) 7:5. 10.3389/fpubh.2019.00005 30733938 PMC6353845

[7] Heine C Browning CJ. Mental health and dual sensory loss in older adults: a systematic review. Front Aging Neurosci. (2014) 6:83. 10.3389/fnagi.2014.00083 24860496 PMC4030176

[8] Heesterbeek TJ van der Aa HPA van Rens G Twisk JWR van Nispen RMA. The incidence and predictors of depressive and anxiety symptoms in older adults with vision impairment: a longitudinal prospective cohort study. Ophthalmic Physiol Opt. (2017) 37:385–98. 10.1111/opo.12388 28516509

[9] Renaud J Bédard E. Depression in the elderly with visual impairment and its association with quality of life. Clin Interv Aging. (2013) 8:931–43. 10.2147/CIA.S27717 23888110 PMC3722036

[10] Simning A Fox ML Barnett SL Sorensen S Conwell Y. Depressive and anxiety symptoms in older adults with auditory, vision, and dual sensory impairment. J Aging Health. (2019) 31:1353–75. 10.1177/0898264318781123 29896982 PMC6274614

[11] Giloyan A Harutyunyan T Petrosyan V. Visual impairment and depression among socially vulnerable older adults in Armenia. Aging Ment Health. (2015) 19:175–81. 10.1080/13607863.2014.920298 24898137

[12] Kiely KM Anstey KJ Luszcz MA. Dual sensory loss and depressive symptoms: the importance of hearing, daily functioning, and activity engagement. Front Hum Neurosci. (2013) 7:837. 10.3389/fnhum.2013.00837 24379769 PMC3864127

[13] Ribeiro MVMR Hasten-Reiter Júnior HN Ribeiro EAN Jucá MJ Barbosa FT Sousa-Rodrigues CF de. Association between visual impairment and depression in the elderly: a systematic review. Arq Bras Oftalmol. (2015) 78:197–201. 10.5935/0004-2749.20150051 26222114

[14] Zhao Y Hu Y Smith JP Strauss J Yang G. Cohort profile: the China Health and Retirement Longitudinal Study (CHARLS). Int J Epidemiol. (2014) 43:61–8. 10.1093/ije/dys203 23243115 PMC3937970

[15] von Elm E Altman DG Egger M Pocock SJ Gøtzsche PC Vandenbroucke JP. The Strengthening the Reporting of Observational Studies in Epidemiology (STROBE) Statement: guidelines for reporting observational studies. Epidemiology. (2007) 18:800–4. 10.1097/EDE.0b013e3181577654 18049194

[16] Uchino M Kawashima M Kaido M Suwaki K Uchino Y Kawachi I. Evaluation of a paper-based visual acuity questionnaire. Clin Ophthalmol. (2017) 11:1213–7. 10.2147/OPTH.S138399 28721005 PMC5499935

[17] Xie T Liu D Guo J Zhang B. The longitudinal effect of sensory loss on depression among Chinese older adults. J Affect Disord. (2021) 283:216–22. 10.1016/j.jad.2021.01.081 33561802

[18] Phua J Visaria A Østbye T Malhotra R. Association of vision and hearing impairments with quality of life among older adults: Mediation by psychosocial factors. Geriatr Gerontol Int. (2022) 22:56–62. 10.1111/ggi.14318 34852404

[19] Andresen EM Malmgren JA Carter WB Patrick DL. Screening for depression in well older adults: evaluation of a short form of the CES-D. Am J Prev Med. (1994) 10:77–84. 10.1016/S0749-3797(18)30622-6 8037935

[20] Li H Zheng D Li Z Wu Z Feng W Cao X. Association of depressive symptoms with incident cardiovascular diseases in middle-aged and older Chinese adults. JAMA Netw Open. (2019) 2:e1916591. 10.1001/jamanetworkopen.2019.16591 31800066 PMC6902756

[21] Chen H Mui AC. Factorial validity of the Center for Epidemiologic Studies Depression Scale short form in older population in China. Int Psychogeriatr. (2014) 26:49–57. 10.1017/S1041610213001701 24125553

[22] Bernabei V Morini V Moretti F Marchiori A Ferrari B Dalmonte E. Vision and hearing impairments are associated with depressive–anxiety syndrome in Italian elderly. Aging Ment Health. (2011) 15:467–74. 10.1080/13607863.2011.562483 21500013

[23] Gong R Hu X Gong C Long M Han R Zhou L. Hearing loss prevalence and risk factors among older adults in China. Int J Audiol. (2018) 57:354–9. 10.1080/14992027.2017.1423404 29400111

[24] Lim NCS Fan CHJ Yong MKH Wong EPY Yip LWY. Assessment of depression, anxiety, and quality of life in Singaporean patients with glaucoma. J Glaucoma. (2016) 25:605–12. 10.1097/IJG.0000000000000393 26950574

[25] Grant A Aubin MJ Buhrmann R Kergoat MJ Freeman EE. Visual impairment, eye disease, and the 3-year incidence of depressive symptoms: The Canadian Longitudinal Study on Aging. Ophthalmic Epidemiol. (2021) 28:77–85. 10.1080/09286586.2020.1823425 32970494

[26] Zheng DD Bokman CL Lam BL Christ SL Swenor BK West SK. Longitudinal relationships between visual acuity and severe depressive symptoms in older adults: the Salisbury Eye Evaluation study. Aging Ment Health. (2016) 20:295–302. 10.1080/13607863.2015.1008985 25673222 PMC4534361

[27] Frank CR Xiang X Stagg BC Ehrlich JR. Longitudinal associations of self-reported vision impairment with symptoms of anxiety and depression among older adults in the United States. JAMA Ophthalmol. (2019) 137:793–800. 10.1001/jamaophthalmol.2019.1085 31095253 PMC6537761

[28] Ye X Zhu D Chen S He P. The association of hearing impairment and its severity with physical and mental health among Chinese middle-aged and older adults. Health Qual Life Outcomes. (2020) 18:155. 10.1186/s12955-020-01417-w 32456646 PMC7249342

[29] Kim SY Kim H-J Park E-K Joe J Sim S Choi HG. Severe hearing impairment and risk of depression: a national cohort study. PLoS One. (2017) 12:e0179973. 10.1371/journal.pone.0179973 28640916 PMC5481021

[30] Han JH Lee HJ Jung J Park EC. Effects of self-reported hearing or vision impairment on depressive symptoms: a population-based longitudinal study. Epidemiol Psychiatr Sci. (2019) 28:343–55. 10.1017/S2045796018000045 29415786 PMC6998913

[31] Maaswinkel IM van der Aa HPA van Rens GHMB Beekman ATF Twisk JWR van Nispen RMA. Mastery and self-esteem mediate the association between visual acuity and mental health: a population-based longitudinal cohort study. BMC Psychiatry. (2020) 20:461. 10.1186/s12888-020-02853-0 32972387 PMC7513319

[32] Lisan Q van Sloten TT Lemogne C Offredo L Climie RE Boutouyrie P. Association of hearing impairment with incident depressive symptoms: a community-based prospective study. Am J Med. (2019) 132:1441–9.e4. 10.1016/j.amjmed.2019.05.039 31247178

[33] Carrière I Delcourt C Daien V Pérès K Féart C Berr C. prospective study of the bi-directional association between vision loss and depression in the elderly. J Affect Disord. (2013) 151:164–70. 10.1016/j.jad.2013.05.071 23787409

[34] Hong T Mitchell P Burlutsky G Gopinath B Liew G Wang JJ. Visual impairment and depressive symptoms in an older Australian cohort: longitudinal findings from the Blue Mountains Eye Study. Br J Ophthalmol. (2015) 99:1017–21. 10.1136/bjophthalmol-2014-306308 25722491

[35] Tetteh J Fordjour G Ekem-Ferguson G Yawson AO Boima V Entsuah-Mensah K. Visual impairment and social isolation, depression and life satisfaction among older adults in Ghana: analysis of the WHO's Study on global AGEing and adult health (SAGE) Wave 2. BMJ Open Ophthalmol. (2020) 5:e000492. 10.1136/bmjophth-2020-000492 32626826 PMC7326267

[36] Coyle CE Steinman BA Chen J. Visual acuity and self-reported vision status: their associations with social isolation in older adults. J Aging Health. (2017) 29:128–48. 10.1177/0898264315624909 26762396 PMC4940280

[37] Toyoshima A Martin P Sato S Poon LW. The relationship between vision impairment and well-being among centenarians: findings from the Georgia Centenarian Study. Int J Geriatr Psychiatry. (2018) 33:414–22. 10.1002/gps.4763 28741698

[38] Zhang X Bullard KM Cotch MF Wilson MR Rovner BW McGwin G Jr. Association between depression and functional vision loss in persons 20 years of age or older in the United States, NHANES 2005-2008. JAMA Ophthalmol. (2013) 131:573–81. 10.1001/jamaophthalmol.2013.2597 23471505 PMC3772677

[39] Dong X Ng N. Contribution of multiple pathways to the relationship between visual impairment and depression: explaining mental health inequalities among older Chinese adults. J Affect Disord. (2021) 278:350–6. 10.1016/j.jad.2020.09.068 33002726

[40] Choi JS Betz J Li L Blake CR Sung YK Contrera KJ. Association of using hearing aids or cochlear implants with changes in depressive symptoms in older adults. JAMA Otolaryngol Head Neck Surg. (2016) 142:652–7. 10.1001/jamaoto.2016.0700 27258813 PMC12861333

[41] Man REK Gan ATL Fenwick EK Gupta P Thakur S Fang XL. The differential impact of age on vision-related quality of life across the visual impairment spectrum. Ophthalmology. (2021) 128:354–63. 10.1016/j.ophtha.2020.07.046 32738259

[42] Brown RL Barrett AE. Visual impairment and quality of life among older adults: an examination of explanations for the relationship. J Gerontol B Psychol Sci Soc Sci. (2011) 66:364–73. 10.1093/geronb/gbr015 21402645

